# Back-to-Back Comparison of Auto-Fluorescence Imaging (AFI) Versus High Resolution White Light Colonoscopy for Adenoma Detection

**DOI:** 10.1186/1471-230X-12-75

**Published:** 2012-06-22

**Authors:** Kentaro Moriichi, Mikihiro Fujiya, Ryu Sato, Jiro Watari, Yoshiki Nomura, Toshie Nata, Nobuhiro Ueno, Shigeaki Maeda, Shin Kashima, Kentaro Itabashi, Chisato Ishikawa, Yuhei Inaba, Takahiro Ito, Kotaro Okamoto, Hiroki Tanabe, Yusuke Mizukami, Yusuke Saitoh, Yutaka Kohgo

**Affiliations:** 1 Division of Gastroenterology and Hematology/Oncology, Department of Medicine, Asahikawa Medical University, 2-1 Midorigaoka-higashi, Asahikawa, Hokkaido, 078-8510, Japan; 2 Internal Medicine, Kotoni Royal Hospital, 2-Nishi1-1-1, Hachiken, Nishi-ku, Sapporo, Hokkaido, 063-0842, Japan; 3 Internal Medicine, Kushiro Medical Association Hospital, 4-30 Nusamai-cho, Kushiro, Hokkaido, 085-0836, Japan; 4 Division of Upper Gastroenteroelogy, Department of Internal Medicine, Hyogo College of Medicine, 1-1 Mukogawa-cho, Nishinomiya, Hyogo, 663-8501, Japan; 5 Digestive Disease Center, Asahikawa City Hospital, 1-1-65 Kinsei-cho, Asahikawa, Hokkaido, 070-8610, Japan

**Keywords:** Autofluorescence imaging, Colorectal adenoma, Detection rate, Flat and depressed adenoma, Less-experienced endoscopist, High-resolution colonoscope

## Abstract

**Background:**

Some patients under close colonoscopic surveillance still develop colorectal cancer, thus suggesting the overlook of colorectal adenoma by endoscopists. AFI detects colorectal adenoma as a clear magenta, therefore the efficacy of AFI is expected to improve the detection ability of colorectal adenoma. The aim of this study is to determine the efficacy of AFI in detecting colorectal adenoma.

**Methods:**

This study enrolled 88 patients who underwent colonoscopy at Asahikawa Medical University and Kushiro Medical Association Hospital. A randomly selected colonoscopist first observed the sigmoid colon and rectum with conventional high resolution endosopy (HRE). Then the colonoscopist changed the mode to AFI and handed to the scope to another colonoscopist who knew no information about the HRE. Then the second colonoscopist observed the sigmoid colon and rectum. Each colonoscopist separately recorded the findings. The detection rate, miss rate and procedural time were assessed in prospective manner.

**Results:**

The detection rate of flat and depressed adenoma, but not elevated adenoma, by AFI is significantly higher than that by HRE. In less-experienced endoscopists, AFI dramatically increased the detection rate (30.3%) and reduced miss rate (0%) of colorectal adenoma in comparison to those of HRE (7.7%, 50.0%), but not for experienced endoscopists. The procedural time of HRE was significantly shorter than that of AFI.

**Conclusions:**

AFI increased the detection rate and reduced the miss rate of flat and depressed adenomas. These advantages of AFI were limited to less-experienced endoscopists because experienced endoscopists exhibited a substantially high detection rate for colorectal adenoma with HRE.

## Background

Colorectal cancer is one of the most common malignant tumors in Eastern and Western countries [1]. During the process of colon carcinogenesis, normal epithelia are thought to initially turn into benign adenomas, accumulate gene alterations and then transform to advanced adenocarcinomas [2][3]. All adenoma are considered to be premalignant lesions. The elimination of colon adenoma is therefore an effective strategy to prevent the development of colon cancer. Several trials on an endoscopic resection for colon adenoma successfully decreased the mortality of colon cancer [4]. However, some patients under close colonoscopic surveillance still develop colorectal cancer [5]. This discrepancy may be caused by the rapid progression of adenomas as well as the overlooking of colorectal adenoma. Indeed, systematic reviews of back-to back colonoscopies showed that 15% to 32% of colorectal adenomas were possibly missed by colonoscopy [6], particularly flat and depressed adenomas [7][8][9][10]. Advanced endoscopic instruments may therefore decrease the miss rate of adenomas and optimize the potential for colorectal cancer prevention.

AFI is a novel technology which can capture fluorescence (500–630 nm) emitted from intestinal or other tissues. This device produces an excitation light source of 442 nm, delivers it to the tissue surface and then captures the reflection and fluorescence by two high-sensitivity CCDs. Those captured signals are respectively transformed into red or blue colors, and then are displayed on the monitor as a color image in real-time [11][12]. Fluorescence imaging is thought to be an efficient tool for the evaluation of dysplasia in Barrett esophagus [13][14][15], esophageal and gastric cancer [16] and dysplasia [17][18][19], as well as for making a differential diagnosis of intestinal lymphoma [20] and also for assessing the inflammation activity in ulcerative colitis [21]. Three studies proposed the controversial results concerning the usefulness of AFI on the detection of colon lesions. Matsuda et al. proposed the predominance of AFI for detecting polyps of the proximal colon compared to white light endoscopy using a modified back-to-back method [22]. In contrast, the other two studies showed that AFI did not reduce the adenoma miss-rate using two inspections of the entire colon by conventional high-resolution endoscopy (HRE) or AFI [23][24]. There seems to be some bias with regard to these results, since the endoscopic findings of HRE and AFI could not be assessed independently because each colonoscopic examination was performed by a single endoscopist in those studies. Therefore, the efficacy of AFI in detecting colorectal adenoma still remains to be elucidated.

The current study aimed to assess the efficacy of AFI for improving the colorectal adenoma detection rate by comparing experienced and less experienced endoscopists in a prospective manner.

## Methods

### Patients

**Figure 1:**
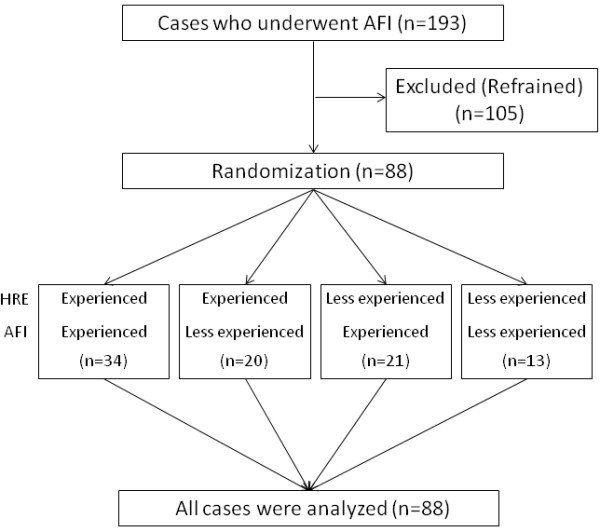
A flow diagram of this study.

This prospective study was registered with University Hospital Medical Information Network (ID number; R000002463). Written informed consent was obtained from all patients enrolled and the study was approved by the institutional review board of Asahikawa Medical University and Kushiro Medical Association Hospital. Eighty-eight patients were enrolled in this prospective study. All patients underwent colonoscopy with an AFI examination (CF-FH260AZI, Olympus medical systems, Tokyo, Japan) at Asahikawa Medical University and Kushiro Medical Association Hospital between January 2008 and December 2008. According to the inclusion criteria, patients who could understand the background information, aims, methods and potential adverse effects of participating in the study were enrolled. The exclusion criteria were an age younger than 18 years, polyposis syndrome, inflammatory bowel disease, severe coagulopathy, stricture, bleeding, and severe cardiac, pulmonary or renal diseases. A total of 193 patients were initially eligible for this study, and 108 of these patients were excluded because they did not consent to participate. Finally, 88 patients were enrolled in this study (Figure 1). The demographics of the enrolled patients and detected lesions are summarized in Table 1. The indications for colonoscopy were abdominal symptoms such as abdominal pain and abnormal defecation in 28 patients, for screening in 38 patients and for surveillance in 22 patients. The shape of the lesions was classified according to the Paris endoscopic classification [25].

**Table 1:** Demographics of the patients and detected lesions


M : F	45 : 43
Mean age (y.o.)	64.0 ± 10.0
Indication for colonoscopy	
Indication for colonoscopy (symptoms: screening: surveillance)	28 : 38 : 22
Number of lesions	88
Adenoma	29
Size (mm)	5.8 ± 6.5
Type 0-IIa	18
Type 0-IIb or 0-IIc	11
Hyperplasia	59
Size (mm)	3.7 ± 1.8
Elevated	13
Flat & depressed	46

### Colonoscopic instruments

**Figure 2:**
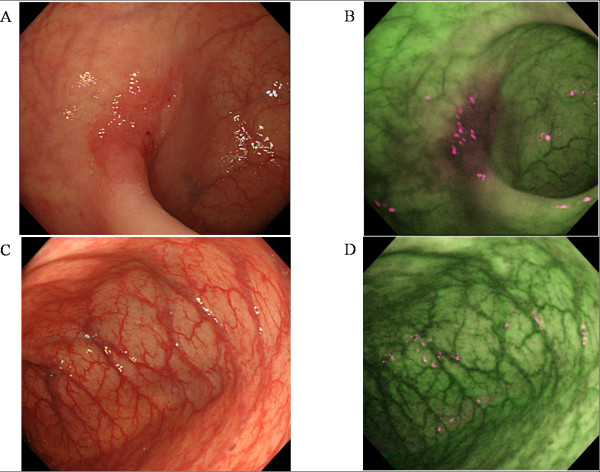
**Cases of flat and depressed adenoma and normal mucosa.** A faint red area in the sigmoid colon was noted by HRE (**A**). AFI revealed a strong magenta area at the same site (**B**). In the normal mucosa, HRE showed no abnormal findings (**C**) and AFI revealed no magenta area (**D**).

A high definition colonoscope (CF-FH260AZI; Olympus Inc.) containing 2 CCDs: 1 for HRE/NBI and 1 for AFI, an Olympus Lucera Spectrum video processor and a high definition monitor were used for the colonoscopy studies. AFI uses blue light (390 –470 nm) for excitation and green light (540–560 nm) is for reflection. A barrier filter allows passage of light to the charge-coupled devices with wavelengths between 500 and 630 nm only, and consisting of autofluorescence emission and green reflectance. AFI images were diagnosed based on the predominant color intensity. The normal mucosa and adenoma appear green and magenta, respectively, by AFI (Figure 2).

### Colonoscopic procedure and randomization

All patients were prepared using 2 L of polyethylene glycol solution in this study. None of them exhibited insufficient bowel preparation. All procedures were performed by one of either 5 experienced endoscopists (> 8000 standard and > 100 AFI) or 3 less-experienced endoscopists (< 500 standard and < 10 AFI). Because this study aimed to elucidate the differences in the significance of AFI between experienced and less-experienced endoscopists, we selected highly experienced endoscopists and endoscopists with much less experience. A colonoscopist who was randomly selected with a sealed envelope method first observed the sigmoid colon and rectum (for 40 cm from anal verge) with HRE. Next, the colonoscopist changed the mode for AFI and handed to the scope to another colonoscopist who was not aware of the information of HRE. The second colonoscopist who was also selected using a sealed envelope method inserted a colonoscope for 40 cm and observed the sigmoid colon and rectum under sufficient filling with air. Each colonoscopist separately recorded the colonoscopic findings including the location, color and shape of the detected lesions (Figure 1). Thereafter, both colonoscopists cooperated to insert the scope again and obtain biopsy specimens from all detected lesions. The biopsied specimens were histologically diagnosed by a pathologist with no clinical and endoscopic information according to the Vienna classification [26]. The entire procedural time for each procedure was separately measured and recorded for all the patients. The detected lesions were classified into two groups, elevated type (0-IIa) and flat or depressed type (0-IIb or -IIc) according to Paris classification [25]. The detection rate and miss rate of colorectal adenoma by all participants, experienced or less-experienced endoscopists were assessed. The ratio of the cases with adenoma divided by the all cases enrolled was defined as the detection rate. The miss rate was defined as the ratio of all detected adenoma divided by the adenoma as diagnosed by each procedure. The primary goal of this study was to determine the detection ability of AFI in comparison to that of HRE. The second goal was to explore whether the usefulness of AFI for detecting colon lesions was influenced by the morphological features of colon lesions and the experience of the endoscopist. These two secondary outcomes were both exploratory.

### Statistical analysis

To determine the sample size, our preliminary data suggested that the miss rates of HRE and AFI for the detection of colon adenoma were 25.0% and 4.2%, respectively. When the alpha error was defined as 0.05 (two-sided) and the power as 0.8, the number of cases required was estimated to be 66 (33 for each group). Twenty-five percent of the enrolled cases were thought to drop out of the study, so the target number of cases was 85. A total of 88 patients were enrolled and analyzed in this study.

The Wilcoxon signed-rank test was applied for the statistical analysis of the detection and miss rates, and for the procedural time in all participants, because the data were paired and non-parametric. The Mann–Whitney U-test was used to perform the statistical analysis of such data by either experienced or less-experienced endoscopists, because the data were unpaired and non-parametric. A *p* value < 0.05 was considered to be statistically significant. For the purposes of the statistical analysis, individual lesions were assumed to constitute statistically independent observations even when more than one lesion was present in a single patient.

## Results

The detection rate of colorectal adenoma and procedural time of HRE and AFI (Table 2).

**Table 2:** The detection rate of colorectal adenoma w5th HRE and AFI

	**Procedures**	**Examined cases**	**Cases with adenoma detected**	**Detection rates (%)**	**p value**
All adenoma	HRE	88	16	18.2	p < 0.05
	AFI	88	23	26.1	
Flat and depressed adenoma	HRE	88	3	3.4	p < 0.05
	AFI	88	8	9.1	
Elevated adenoma	HRE	88	13	14.8	N.S.
	AFI	88	15	17.0	
Cases examined by experienced endoscopists	HRE	54	14	25.9	N.S.
	AFI	55	13	23.6	
Cases examined by less experienced endoscopists	HRE	34	2	5.9	p < 0.05
	AFI	33	10	30.3	

N.S.; not significant.

HRE was performed by experienced endoscopists in 54 of 88 cases and by less-experienced endoscopists in 34 cases. AFI was performed by experienced endoscopists in 55 cases and by less-experienced endoscopists in 33 cases. In 24 of 88 cases, one or more adenomas, which were histologically classified into category 3 or 4.1 according to the Vienna classification [26], were detected by either HRE or AFI. No lesions corresponding to false positive regions, such as normal mucosa or vascular abnormalities, were detected by either HRE or AFI. Whereas HRE detected one or more colorectal adenomas in 16 of 88 cases (18.2%), AFI detected colorectal adenoma in 23 cases (26.1%). The detection rate for AFI is therefore significantly higher than that for HRE (p < 0.05). AFI detected 8 0-IIb or 0-IIc adenomas in 88 cases while conventional colonoscopy detected only 3 (p < 0.05). In contrast, no difference was observed between HRE and AFI regarding the detection rate of 0-IIa adenomas.

**Figure 3:**
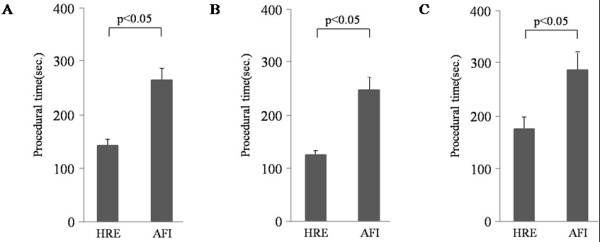
**Procedural time of HRE and AFI.** The procedural time of HRE (144.5 ± 8.8 seconds) was significantly shorter than that of AFI in all participants (267.0 ± 20.8 seconds; p < 0.05) (**A**). An increase in the procedural time for the AFI examination was revealed in either experienced endoscopists (**B**) or less-experienced endoscopists (**C**).

AFI dramatically increased the detection rate of colorectal adenoma (30.3%) in comparison to that of HRE (7.7%; p < 0.05) in less-experienced endoscopists. Conversely, the detection rate of HRE in experienced endoscopists (22.6%) was not significantly different from that of AFI (23.6%). The procedural time of HRE (144.5 ± 8.8 seconds) was significantly shorter than that of AFI in all participants (267.0 ± 20.8 seconds; p < 0.05; Figure 3A). This increase in the length of time required to perform the AFI examination was identified for both experienced endoscopists (Figure 3B) as well as less-experienced endoscopists (Figure 3C).

The miss rate of colorectal adenoma with HRE and AFI (Table 3).

**Table 3:** The miss rate of colorectal adenoma with HRE and AFI

	**lesions**	**Procedures**	**Number of lesions**	**detected**	**missed**	**Miss rates (%)**	**p value**
All participants	Total	HRE	88		38	43.2	p < 0.0001
		AFI	88		4	4.5	
	Adenoma	HRE	29		8	27.6	p < 0.05
		AFI	29		1	3.4	
	Hyperplasia	HRE	59		30	50.8	p < 0.0001
		AFI	59		3	5.1	
Experienced endoscopists	Total	HRE	60		23	38.3	p < 0.0001
		AFI	62		3	4.8	
	Adenoma	HRE	25		6	24.0	N.S.
		AFI	19		1	5.3	
	Hyperplasia	HRE	35		17	48.6	p < 0.0001
		AFI	43		2	4.7	
Less experienced endoscopists	Total	HRE	28		15	53.6	p < 0.0001
		AFI	26		1	3.8	
	Adenoma	HRE	4		2	50.0	p < 0.05
		AFI	10		0	0.0	
	Hyperplasia	HRE	24		13	54.2	p < 0.005
		AFI	16		1	6.3	

N.S.; not significant.

Twenty-nine adenomas were detected by either HRE or AFI in 88 cases. Twenty-one of the 29 adenomas detected from the 88 patients were captured by HRE and 28 were captured by AFI. The miss rate of HRE (27.6%) was significantly higher than that of AFI (3.4%; p < 0.05). The miss rate of HRE (50.0%) was much higher than that of AFI (0%) in less-experienced endoscopists while no significant deference in the miss rate was seen between HRE (24%) and AFI (5.3%) in experienced endoscopists, thus suggesting the efficacy of AFI in detecting colorectal adenoma by less-experienced endoscopists.

## Discussion

The current study demonstrated that AFI improved the detection rate of colorectal adenoma, particularly flat and depressed adenomas, but not for elevated adenomas. An elevated adenoma is easily detected as a reddish and protruding lesion by HRE because HRE can detects abnormalities through capturing the changes of color and shape. However, flat and depressed adenoma is sometimes described just a faint red areas with no elevation, which thus tend to be difficult to detect by HRE. On the other hand, AFI captures the fluorescence mostly emitted from collagen in the submucosal layer and diagnoses the abnormal area through a diminished fluorescence intensity. The dysplastic grade of the lesions is the main factor that affects the fluorescence captured by AFI [27]. The increase in dysplasia appears to facilitate the detection rate of colorectal adenoma by AFI, regardless the shape of the lesion.

A noteworthy finding of the current study is that the efficacy of AFI to detect colorectal adenoma was limited for less-experienced endoscopists. The experienced endoscopists appeared to empirically recognize many of adenomas, even flat and depressed adenoma, with HRE, and thus AFI was not a superior disgnostic modality for experienced endoscopists in detecting colorectal adenoma. The present study revealed that the miss rate with HRE by experienced endoscopists (24.0%) is relatively lower than that previously reported [22][26][28][29][30]. This was probably because only highly experienced endoscopists (> 8000 standard and > 100 AFI) were strictly selected as experienced endoscopists in this study. Conversely, it should not be easy for less-experienced endoscopists to find colorectal adenoma (50.0%), particularly flat and depressed adenoma, with HRE. Most endoscopists, including less-experienced endoscopists, can immediately utilize AFI because AFI diagnosis is simply based on the color intensity of magenta. This objectivity of AFI probably facilitated the detection rate and reduced the miss rate of colorectal adenoma in less-experienced endoscopists. The clinical value of HRE and AFI is therefore considered to depend on the experience of endoscopists.

Three previous reports have shown controversial results concerning the efficacy of AFI for detecting colorectal adenoma. Matsuda et al. concluded that AFI showed a higher detection rate than did white light endoscopy when using a modified back to back method wherein the authors observed the proximal colon twice, once with white light endoscopy and once with AFI, with both examinations being performed by the same endoscopist [22]. Subsequently, two investigations proposed the detection rate of AFI and HRE using the back to back method. These studies inspected the entire colon twice during withdrawal: once with AFI and once with HRE by the same endoscopist and found no efficiency of AFI for reducing the adenoma miss-rate in comparison to HRE [23][24]. These studies possess a potential bias because both endoscopic examinations are performed by the same endoscopist and the second endoscopic diagnosis was therefore influenced by the findings of the first endoscopy. This may explain the controversial results obtained from the two similar studies. Conversely, in the current study, AFI was performed by an endoscopist who was not aware of the information of HRE. This allowed the determination of the efficiency of AFI in the detection of flat and depressed adenoma by less-experienced endoscopists.

Chromoendoscopy can improve the detection of colonic lesions [31-36]. However, chromoendoscopy with magnified observation is time-consuming, operator-dependent and impossible to switch back to the conventional colonoscopy, which may affect the ability to detect mucosal abnormalities in other areas. Narrow band imaging (NBI), a new system in which spectral features are modified by narrowing the bandwidth of spectral transmittance with optical filters and can assess capillary architecture and microvessels just by manipulating a button [37][38], is also a promising tool to improve the detection rate of colorectal adenoma [28][39]. Indeed, recent studies have demonstrated adenoma miss-rates for HRE of 40% to 46% when a second examination was performed with NBI. This suggests that NBI is superior to HRE when utilized for adenoma detection [28][29]. Because NBI has a high ability to discriminate colon adenoma from hyperplasia [40][41], the combination of AFI and NBI can potentially improve the selective detection rate of colon adenoma. Further analysis to compare the efficacy of AFI, NBI or their combination for detecting colon neoplasms is therefore needed to establish the optimal diagnostic modality to perform screening colonoscopy using new advanced imaging systems such as NBI and AFI.

The procedural time to perform AFI (267.0 ± 20.8 seconds) was significantly longer than that of HRE (144.5 ± 8.8 seconds). This is a disadvantage of AFI when using it as screening examinations. Whereas AFI can capture the fluorescence emitted from intestinal tissue, the resolution of AFI is still not sufficient. Therefore, endoscopists have to be cautious when performing colonoscopic examinations with AFI, in order not to increase the overall examination time. New advances in endoscopic tools are expected to produce a higher resolution and optimized operation system of AFI, thus leading to a reduction in the time needed to perform the AFI procedures.

Because it is not easy to judge the lesions detected by both HRE and AFI, or either of the two procedures (particularly when multiple lesions are detected), when the entire colon is targeted, this study investigated only the recto-sigmoid colon. This is thought to be a limitation of this study. A further analysis covering the entire colon will be warranted to draw conclusions about the overall usefulness of AFI for the detection of colon lesions.

## Conclusion

The current study demonstrated that AFI increased the detection rate and reduced the miss rate of colorectal adenoma, particularly flat and depressed adenomas. However, this advantage of AFI was limited to the less-experienced endoscopists because experienced endoscopists exhibited a substantially low miss rate of colorectal adenoma with HRE. Further advances in endoscopic instruments will provide an AFI with a high resolution and optimized operation system, thereby preventing the lengthy procedural time which is a disadvantage of AFI.

## Competing interests

We declare that no authors have any financial relationships with commercial entities producing health-care related products and/or services relevant to this article.

## Author’s contributions

K.M. and M.F. provided major input into the conceptual development of the studies, wrote the manuscript and supervised all investigations. R.S., J.W., Y.N., T.N., N.U., S.K., K.I., Y.I. and H.T. performed the endoscopic examinations. Y.I. assessed the histological findings. S.M., C.I., T.I. and K.O. managed and treated the enrolled patients and collected and analyzed the data. Y.M., Y.S. and Y.K. helped design studies, interpret the data, and prepare/review the manuscript. All authors read and approved the final manuscript.

## Pre-publication history

The pre-publication history for this paper can be accessed here:

http://www.biomedcentral.com/1471-230X/12/75/prepub
